# Molecular pathways of major depressive disorder converge on the synapse

**DOI:** 10.1038/s41380-022-01806-1

**Published:** 2022-10-06

**Authors:** Gabriel R. Fries, Valeria A. Saldana, Johannes Finnstein, Theo Rein

**Affiliations:** 1 Translational Psychiatry Program, Faillace Department of Psychiatry and Behavioral Sciences, The University of Texas Health Science Center at Houston, 1941 East Rd, Houston, TX 77054 USA; 2 Neuroscience Graduate Program, The University of Texas MD Anderson Cancer Center UTHealth Graduate School of Biomedical Sciences, 6767 Bertner Ave, Houston, TX 77030 USA; 3 Frank H. Netter MD School of Medicine at Quinnipiac University, 370 Bassett Road, North Haven, CT 06473 USA; 4 Department of Translational Research in Psychiatry, Project Group Molecular Pathways of Depression, Max Planck Institute of Psychiatry, Kraepelinstr. 10, 80804 Munich, Germany

**Keywords:** Neuroscience, Molecular biology, Depression

## Abstract

Major depressive disorder (MDD) is a psychiatric disease of still poorly understood molecular etiology. Extensive studies at different molecular levels point to a high complexity of numerous interrelated pathways as the underpinnings of depression. Major systems under consideration include monoamines, stress, neurotrophins and neurogenesis, excitatory and inhibitory neurotransmission, mitochondrial dysfunction, (epi)genetics, inflammation, the opioid system, myelination, and the gut-brain axis, among others. This review aims at illustrating how these multiple signaling pathways and systems may interact to provide a more comprehensive view of MDD’s neurobiology. In particular, considering the pattern of synaptic activity as the closest physical representation of mood, emotion, and conscience we can conceptualize, each pathway or molecular system will be scrutinized for links to synaptic neurotransmission. Models of the neurobiology of MDD will be discussed as well as future actions to improve the understanding of the disease and treatment options.

## Introduction and scope

Major depressive disorder (MDD) is a common and (potentially) disabling psychiatric disorder affecting as many as 12% of adults globally, with its prevalence in the United States being highest among young adults, women, and the elderly [1]. MDD represents a major burden on public health, ranking third in the leading causes of disability worldwide [2], with studies predicting a significant increase of MDD cases globally after the Covid-19 pandemic [3].

The diagnosis of MDD, according to the Diagnostic and Statistical Manual of Mental Disorders, 5th edition, is characterized by 2 or more weeks of depressed mood and/or loss of interest and pleasure, along with other symptoms including sleep, weight, and energy changes [4]. Treatment with antidepressants is often indicated, although ~50% of patients do not achieve remission with first-line treatment [5]. This indicates the need for the development of more effective treatments based on an in-depth understanding of MDD’s pathophysiology.

Over the recent years, neuroimaging studies have identified structural and functional brain changes in patients with MDD. These include volume reductions in cortical and subcortical structures [6, 7], reduced gray matter volume throughout the brain, enlarged lateral ventricles, and white matter microstructural differences suggestive of compromised myelin integrity [6, 8, 9]. In parallel, postmortem studies have reported changes in the density and size of neurons and glia in several brain regions of patients [10] along with reduced expression of pre- and postsynaptic genes [11, 12].

**Fig. 1:**
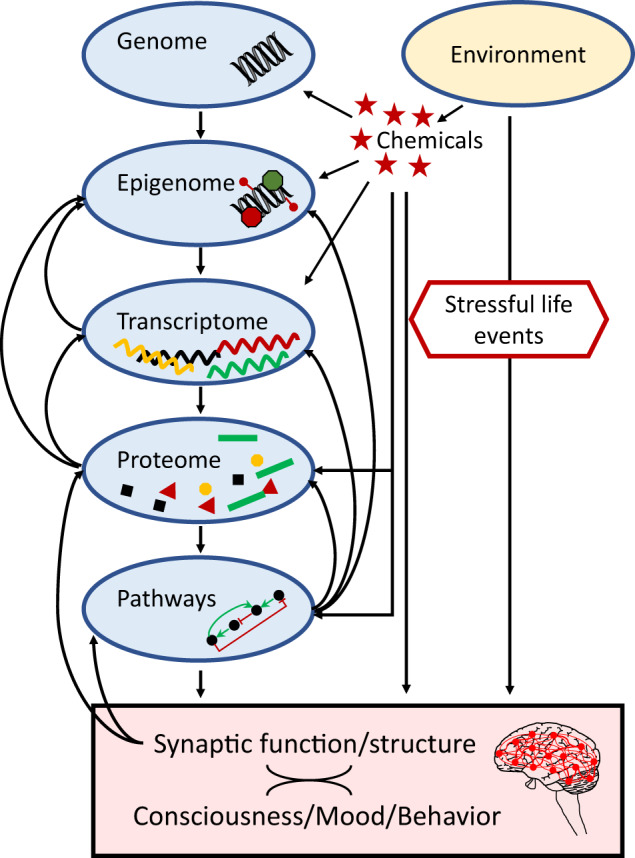
Simplified scheme of the molecular basis of consciousness/depression. The pattern of synaptic activity is regarded as the closest correlate or representation of consciousness and mood, and thus also depression. This review describes selected pathways with established links to depression with a focus on their links to synaptic activity as well as their interrelatedness.

The attempt to understand MDD inadvertently brings up the question of how to comprehend consciousness. Despite the epistemological limitations, and irrespective of the differences between the naturalist theories of consciousness [13, 14], the essential role of synaptic activity in giving rise to higher network pathways from which cognitive, emotional, and behavioral functions emerge is undisputed. Thus, by accepting synaptic activity, or the pattern thereof, as the molecular description that comes closest to consciousness, mood, and depression, the molecular pathways selected for this review will be presented including their proven or potential links to synaptic events (Fig. 1). We will point to the manifold interrelations between these pathways and conclude with discussing examples of integrated models for the molecular underpinnings of MDD and suggestions for future research.

## Molecular pathways and systems

### Genetics and epigenetics

Family, twin, and adoption studies document a complex genetic basis of MDD [15–17]. MDD features a highly polygenic form of inheritance, with multiple loci of small effect size interacting with each other and with environmental triggers. The largest genome-wide association study (GWAS) of depression to date, which included over 1.2 million participants [18], identified 178 genetic risk loci and 223 independent SNPs associated with MDD. The SNP-based heritability for MDD was identified to be around 11.3%, and top biological processes included nervous system development, brain volume, and synapse assembly and function (Table 1) [18].

**Table 1:** Key genes linked to major depressive disorder in the most recent genome-wide association study [18].

Gene	Known functions and roles	Representative impacted tissue
*NEGR1*	Brain volume (hippocampus); social behavior and non-social interest; depressive- and anxiety-like behavior	Hypothalamus
*DRD2*	Reward; depressive-like behavior	Nucleus accumbens
*CELF4*	Sodium channel function; developmental disorders	Caudate
*CCDC71*	Cellular lipid metabolism and regulation of fat cell differentiation [predicted]	Amygdala
*FADS1*	Fatty acid regulation	Cerebellum
*SPPL3*	Cellular glycosylation processes	Prefrontal cortex (BA9)
*TRAF3*	Control of type-1 interferon response	Hypothalamus
*LAMB2*	Cellular adhesion; embryonic development	Blood

*CCDC71* Coiled-Coil Domain Containing 71, *CELF4* CUGBP Elav-like family member 4, *DRD2* Dopamine Receptor D2, *FADS1* Fatty acid desaturase 1, *LAMB2* Laminin Subunit Beta 2, *NEGR1* Neuronal growth regulator 1, *SPPL3* Signal peptide peptidase-like 3, *TRAF3* TNF receptor-associated factor 3.

Top MDD-associated genes in the latest GWAS study are linked to synaptic function: the neuronal growth regulator 1 (*NEGR1*) controls synapse number and dendritic maturation [19]. *NEGR1* SNPs have also been associated with low white matter integrity [20] and responsiveness to selective serotonin (5-HT) reuptake inhibitors [21]. The dopamine D2 receptor (*DRD2*) regulates synaptic pruning and long-term depression through activation of the mammalian target of rapamycin (mTOR) [22]. Finally, CUGBP Elav-Like Family Member 4 (*CELF4*) is a neuronal RNA-binding protein that targets genes associated with the regulation of neuronal excitation, synaptic plasticity, and transmission [23]. CELF4 levels were recently shown to be decreased in an animal model of depression along with decreased spine number [24].

Assuming genetic variants as the “first hits” in a multifactorial disease model, assessing the top genes associated with MDD offers valid biological insight into its onset. Combined with environmental stressors, these variants may induce alterations of small effect at the cellular and physiological level, and may ultimately increase the individual’s vulnerability to future stressful events. Epigenetic regulation of gene activity has been recognized as a key mechanism conveying the lasting molecular impact of these stressors. Many epigenetic alterations in MDD, including DNA methylation, map to genes involved in neuronal circuitry formation, projection, functioning, and plasticity [25–27]. Examples include hypermethylation of the histone deacetylase 4 gene, in line with its role in neuronal morphology and dendritic arborization [28], hippocampal-dependent learning and memory, and long-term synaptic plasticity [29]. Other epigenetic mechanisms include non-coding RNAs [30, 31] and histone modifications [32]. For instance, miR-132, one of the top-ranked upregulated miRNAs in MDD across multiple studies [33], is a regulator of synaptic proteins [34] and synaptic plasticity [35]. Inhibiting miR-132 improves depressive-like symptoms and upregulates brain-derived neurotrophic factor (BDNF) expression in animal models [33].

### The monoamine theory

One of the first suggested biological mechanisms underlying MDD is the deficiency in monoamine levels, i.e., 5-HT, noradrenaline, and dopamine [36]. This “monoamine theory of depression” was supported by initial findings that monoamine oxidase inhibitors and tricyclic antidepressants could improve depressive symptoms by potentiating 5-HT and noradrenaline activity. While many studies later supported this theory, limitations include the fact that the clinical effects of antidepressant treatments typically take weeks to be observed, while the effects of antidepressants to increase monoamine levels are almost instantaneous. Moreover, around one third of depressed patients do not respond to antidepressants that work exclusively by inhibiting monoamine reabsorption, and restricting the availability of the 5-HT precursor tryptophan does not induce depressive episodes in all patients [37]. Thus, monoamine deficiency may not be universal across all patients, pointing to the relevance of other pathways and neurotransmitters for MDD.

#### Other neurotransmitters

MDD is associated with disturbances in other neurotransmitters in the brain, cerebrospinal fluid, and in peripheral tissues [38], including the gamma-aminobutyric acid (GABA) and glutamatergic systems [39, 40]. Glutamate levels are decreased in specific brain regions of patients [41] possibly linked to a decreased response to emotional stimuli and supported by postmortem findings of reduced number of synapses [41]. Thus, newly developed antidepressant treatments focus on reversing glutamate and GABA deficits by addressing glutamate α-Amino-3-hydroxy-5-methyl-4-isoxazolepropionic acid (AMPA) receptors or group 2 metabotropic glutamate receptors [41]. This also led to the discovery of fast-acting antidepressants such as ketamine, which rapidly increases glutamate signaling and leads to rapid and sustained antidepressant response in both preclinical and clinical studies [41]. Mechanistically, ketamine blocks N-methyl-D-aspartate (NMDA) receptor channels and thus excitatory glutamate signaling in GABAergic neurons, increasing the overall activity of the prefrontal cortex. Additionally, ketamine enhances the mTOR complex 1 signaling and increases the number and function of synapses in the prefrontal cortex [42] independently of NMDA receptor inhibition [43]. Further drug developments include brexanolone, an analog of the neurosteroid THP, for the treatment of postpartum depression, as THP levels drop after pregnancy [44]. THP also affects the hypothalamus-pituitary-adrenal (HPA) axis as a positive allosteric modulator for specific subunits of extrasynaptic GABA_A_ receptors (GABAARs) expressed in the paraventricular nucleus of the hypothalamus [40].

#### Relation to other pathways, opioid signaling

**Fig. 2:**
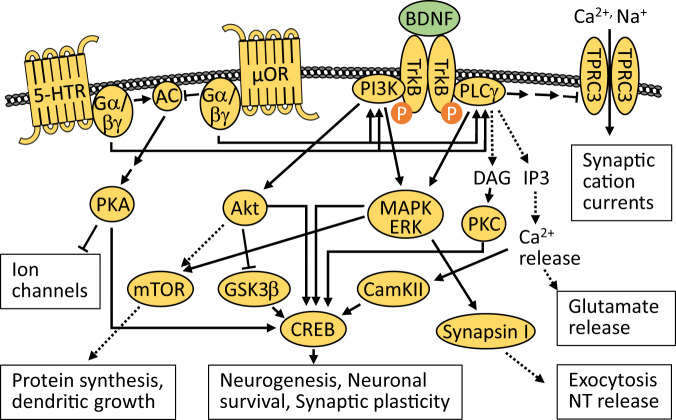
Signaling pathways through the receptors of serotonin, opioids, and BDNF alter neuronal and synaptic functions. Signaling through a variety of receptors is highly intertwined and may produce significantly overlapping effects on neurogenesis, neuronal structure, and synaptic activity. Activation of these pathways may be imbalanced in major depressive disorder (MDD), and at least partly restored by pharmacological treatments targeting various facets of these pathways. AC adenyl cyclase, Akt protein kinase B, BDNF brain-derived neurotrophic factor, CamKII Ca^2+^/Calmodulin-dependent protein kinase II, CREB cAMP responsive element binding protein, DAG diacylglycerol, ERK extracellular signal-regulated kinase, Gα/βγ G-protein subunits α/βγ, GSK3β glycogen synthase kinase 3β, 5-HTR serotonin receptor, IP3 inositol 1,4,5-trisphosphate, MAPK mitogen-activated protein kinase, µOR µ-opiod receptor, mTOR mammalian target of rapamycin, NT neurotransmitters, PI3K phosphoinositide-3-kinase, PKA protein kinase A, PLCγ phospholipase C γ, TrkB tyrosine receptor kinase B, TRPC3 transient receptor potential canonical subfamily 3.

Monoamines not only directly influence synaptic neurotransmission, but also indirectly by affecting intracellular pathways through their G-protein coupled receptors. Protein examples of these pathways include phosphatidylinositol 3-kinase (PI3K), protein kinase C (PKC), Akt, mitogen-activated protein kinase (MAPK), and extracellular signal-regulated kinase (ERK). Of the other receptors addressing these pathways, we focus on the opioid receptors, as they functionally interact with 5-HT and dopamine receptors through heterodimerization [45]. In general, opioid receptors negatively regulate neurotransmitter release and excitability of neurons by the activation of G-protein-mediated mechanisms, resulting in increased potassium channel functioning, cell depolarization, and inhibition of functioning voltage-gated calcium channels, negatively regulating neurotransmitter release [46]. Processes further downstream affect neuronal survival and plasticity [47] (Fig. 2).

Excellent reviews summarize the vast clinical and preclinical evidence for the involvement of opioid receptors in MDD pathology [47]. Examples are the regulation of social bonding, appetite, anhedonia, reward processing, mood and anxiety, and behavior in experimental paradigms of social acceptance/rejection or sustained sadness challenge [48–50]. The opioid dysregulation hypothesis of MDD has sparked intense and promising efforts to develop opioid tone modulating drugs as novel antidepressants [47].

### Neurotrophins

The “neurotrophic hypothesis of depression” stipulates disrupted neurotrophic support as the key mechanism underlying MDD-related synaptic and brain-related alterations. Neurotrophins are growth factors responsible for the formation, support, and plasticity of neuronal networks. BDNF is a prominent member of the large neurotrophin family, which can activate tropomyosin-related kinase (Trk) and p75 receptors. Ample evidence documents altered neurotrophin levels in patients, particularly reduced blood BDNF levels in acute MDD [51] in persistently depressed and remitted patients, [52] and in animal models of depression [53]. Of note, BDNF levels increase after antidepressant treatment and electroconvulsive therapy [51], and higher BDNF levels are associated with better cognitive performance in both patients and controls [54]. Finally, not only is BDNF expression and its downstream signaling required for the action of conventional and rapid-acting antidepressants [55], but a recent study has also found that antidepressants can bind directly to the transmembrane domain of TrkB dimers, rendering a stable conformation of the multi-protein complex and overall promotion of signaling and TrkB accessibility to BDNF [56]. In fact, point mutations in the TrkB transmembrane region have blocked the effects of typical and fast-acting antidepressants [56].

#### Link to synaptic activity

Through Trk receptors, neurotrophins can activate cell signaling pathways controlling cell fate decisions, axonal growth, dendritic growth and pruning, and overall normal neuronal function, including Ras, PI3K, and phospholipase C-γ (PLC-γ) (Fig. 2). BDNF-TrkB signaling also generates sustained synaptic cation currents by activating transient receptor potential canonical subfamily (TRPC) 3 [57]. Furthermore, BDNF-TrkB-PLC-γ signaling via inositol 1,4,5-trisphosphate (IP_3_) induces the release of Ca^2+^ from presynaptic intracellular stores, increasing the number of docked synaptic vesicles and enhancing glutamate release [58]. Another direct link to presynaptic glutamate release operates via the BDNF-TrkB-MAPK/ERK-mediated phosphorylation of synapsin I, thereby facilitating exocytosis and neurotransmitter release [59, 60].

#### Relation to other pathways, neurogenesis

Neurotrophins are intertwined with other depression-related pathways: BDNF is a downstream target of the monoamine signaling cascade [53] (cf. “The monoamine theory”). By activating TrkB receptors, BDNF modulates MAPK/ERK and PI3K/Akt pathways [61], ultimately contributing to impairments in neuronal plasticity and survival. Indeed, reduced levels of ERK and the activity of Akt have been found in postmortem brains of depressed patients [62, 63]. BDNF also activates the mTOR pathway, promoting protein synthesis in neuronal dendrites [64] and regulating the expression of AMPA receptor subunits [65].

One of the most remarkable effects of BDNF is the facilitation of adult neurogenesis in the hippocampus [66], likely operating through most of the above-mentioned signaling [67]. Hippocampal neurogenesis deficits in MDD are implicated by postmortem findings of decreases in hippocampal size and volume, in the number of neurons and glial cells, and in cell size [66]. There is also evidence of a significant interconnectedness between neurogenesis and synaptic activity, including long-term potentiation (LTP) [68]. Adult-born neurons can modulate spine density and excitatory synaptic transmission to existing neurons by redistributing pre-existing synapses [69]. Importantly, antidepressants induce neurogenesis, increase the potential for plasticity, and reverse hippocampal atrophy [66, 67].

### Stress

Stress exposure, particularly early in life, arguably is the best-studied and established risk factor for MDD [70]. Many of the MDD symptoms have been linked to chronic stress, and numerous studies document structural changes of neuronal architecture and function upon stress exposure [71].

The HPA axis is key to orchestrating the organism’s stress response. Crucial to the stress response is its adequate termination through a negative feedback mechanism executed by the stress-secreted glucocorticoids that activate glucocorticoid receptors (GRs). While fast-acting mechanisms of glucocorticoids via membrane receptors have been reported, their bulk and lasting effects operate through nuclear receptors that function as ligand-activated transcription factors with a wide range of effects in several organs [72–74]. This links glucocorticoids to molecular mechanisms of chronic stress as well as early-life stress, including reprogramming of the transcriptome through epigenetic mechanisms [75–78]. Of note, GR not only drives epigenetic writing, but is also subjected to epigenetic programming [78, 79].

Increased cortisol levels, HPA overactivity, and a dysfunctional negative feedback of the HPA axis have been reported in some depressed patients, particularly in specific depression subtypes [80]. Thus, multiple drugs targeting the stress system have been tested for the treatment of depression, including corticosteroid synthesis inhibitors, GR antagonists, corticotrophin-releasing hormone receptor antagonists, tryptophan 2,3-dioxygenase inhibitors, and FK506-binding protein 51 (FKBP51) receptor antagonists [81]. Since not all patients present with alterations in the HPA axis, genetic or functional assessments at baseline for the identification of potentially responsive patients may be required [81]. Indeed, treatment with mifepristone (a GR antagonist) has shown promising results in patients with psychotic depression [82].

#### Link to synaptic activity

The direct effect of stress and chronic exposure to glucocorticoids on functional and structural connectivity is supported by evidence of stress-induced atrophy-like effects on apical dendrites and postsynaptic dendritic spines in the brain [83], resulting in significant synaptic remodeling. Mechanistically, non-genomic actions of glucocorticoids through putative membrane receptors have been invoked, e.g., to contribute to the increase of the readily releasable pool of glutamate vesicles in the prefrontal cortex [84]. Genomic actions are involved in the acute effects of stress and glucocorticoids on the GR-dependent enhanced surface expression of NMDA and AMPA receptors [85]: glucocorticoids transcriptionally activate serum- and glucocorticoid-inducible kinase (SGK) [86] which is required for stress- or glucocorticoid-enhanced activity of Rab4 [84]. Rab4 is a small GTPase that regulates recycling from early endosomes to the cell surface [87] and thus also controls NMDAR and AMPAR recycling [85]. SGK1 has been further linked to MDD through its impact on hippocampal neurogenesis and as an upstream regulator of GR [88]. Finally, the effects of chronic glucocorticoids on dendritic atrophy have also been linked to excessive PKC signaling and reduced expression of neural cell adhesion molecules [83], in addition to suppression of BDNF signaling (“Relation to other pathways”).

Cell-type specific effects include the reduced tonic inhibition in the granule of the dentate gyrus upon chronic stress, likely through reduced expression of the GABAAR δ-subunits and association with impairment in learning and memory, in addition to stress-related depressive-like behavior [89]. Further, microglia are increasingly highlighted for their role in mediating the effects of stress on synaptic structure and function including synaptic pruning and spine density [90, 91]. Notably, microglia activity integrates input from several other sources, not only the neuroendocrine and noradrenergic system, but also cytokines and inflammation, the gut-brain axis, and neurotransmitters [91]. Synaptic and behavioral effects of stress are also mediated, at least partly, through the opioid system; this is largely based on pharmacological and genetic manipulation in animal models and awaits elucidation of further mechanistic details [92, 93].

#### Relation to other pathways

**Fig. 3:**
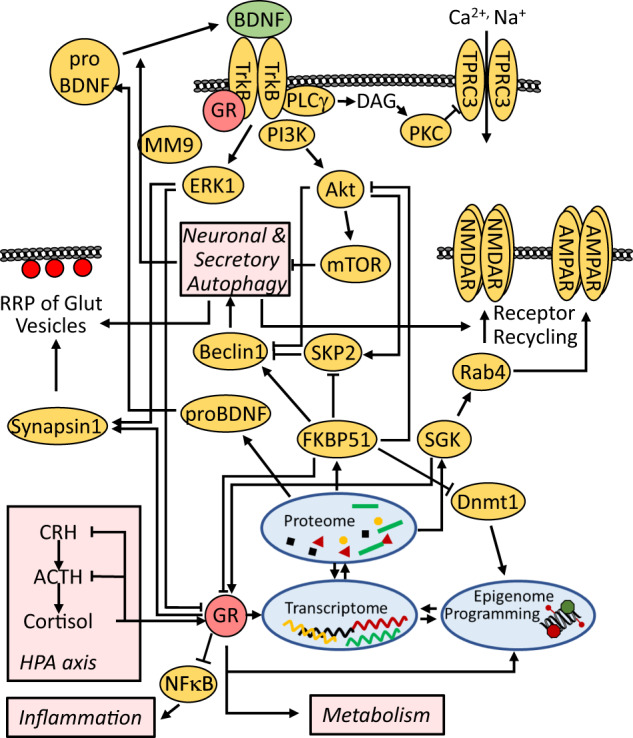
Stress signaling is interwoven with numerous depression-relevant pathways. The levels of the stress hormone cortisol are set by the hypothalamus-pituitary-adrenal (HPA) axis. They activate GR, which exerts both genomic and non-genomic actions. Of the GR-regulated genes, BDNF, FKBP51 and SGK are displayed, including examples of their links to various pathways, physiological systems (metabolism, inflammation, neuronal and secretory autophagy and HPA axis) and activity of synaptic proteins (receptor recycling, vesicle docking and recycling, ion channels). ACTH adrenocorticotropic hormone, AMPAR α-amino-3-hydroxy-5-methyl-4-isoxazolepropionic acid receptor, BDNF brain-derived neurotrophic factor, CRH corticotropin-releasing hormone, Dnmt1 DNA methyltransferase 1, ERK extracellular regulated kinase, FKBP FK506-binding protein, GR glucocorticoid receptor, MM9 matrix metalloproteinase 9, mTOR mammalian target of rapamycin, NFκB nuclear factor kappa B, NMDAR N-methyl-D-aspartate receptor, PI3K phosphatidylinositol 3-kinase, PKC protein kinase C, PLC phospholipase C, Rab ras related protein, RRP readily releasable pool, SGK serum/glucocorticoid regulated kinase, TrkB tropomyosin-related kinase B, TRPC3 transient receptor potential channel 3.

Signal transduction of glucocorticoids is intertwined with most pathways linked to depression (Fig. 3). As examples, we discuss BDNF, FKBP51, and autophagy. BDNF signaling is interrelated with glucocorticoid signaling in multiple ways. Chronic glucocorticoid exposure reduces BDNF mRNA- and protein-levels, its receptor TrkB, and downstream proteins [58]. In contrast, acute effects of glucocorticoids activate the BDNF-TrkB pathway [58]; this divergence between acute and chronic glucocorticoid effects is a recurrent motif in the stress response. Direct protein interaction between GR and TrkB promotes BDNF-TrkB signaling and is diminished by the decreased levels of GR upon chronic stress [94]. GR not only impacts BDNF signaling, but is also modulated by BDNF activity: activation of ERK1 and c-Jun N-terminal kinase downstream of BDNF-TrkB leads to phosphorylation of GR at several sites [95]. Interestingly, these phosphorylation sites are required for the reversal of dendritic spine density loss by fluoxetine in the chronic unpredicted stress model [95].

The GR target gene *FKBP5* emerged as a significant player in depression originally inspired by the inhibitory action of its protein FKBP51 on GR [96–98]. *FKBP5* polymorphisms have been associated with HPA axis parameters, antidepressant treatment response, and recurrence of depressive episodes [99]. FKBP51 is intertwined with other pathways related to depression: it is a target of epigenetic programming [100, 101], but potentially also a sculptor of the epigenetic landscape through its action on DNA methyltransferase 1 [102]. Through additional protein-protein interactions that recalibrate protein phosphorylation, FKBP51 also impacts signaling of other depression-relevant pathways such as GSK3β [103], BDNF [102, 104], and nuclear factor kappa B, linking it to inflammation and the immune system [105, 106], as well as autophagy [107, 108].

Finally, glucocorticoids are linked to autophagy in many ways [109–111]. The importance of autophagy in depression is largely supported by two observations: several antidepressants induce autophagy, and autophagy shapes synaptic neurotransmission and (depressive-like) behavior [108, 112–114]. While most of the evidence is based on cell and animal models, it has been reported that the success of antidepressant treatment of MDD patients correlates with the expression of autophagic markers in blood cells and with the response of these markers to treatment [107]. In general, autophagy is an evolutionarily conserved intracellular degradative process that promotes the homeostasis of energy, proteins, and organelles [115]. It continuously operates at a basal level and is enhanced under various stressful conditions [116]. Several regulatory components of the autophagy cascade are MDD-related. Examples are FKBP51 [107, 117], BDNF [104, 118], and the protein kinases Akt1, mTOR, PI3K, and GSK3β [119]. The relevance of these proteins to MDD is substantiated by their impact on synaptic neurotransmission [59, 120–123]. Therefore, the question arises whether they are relevant to depression due to their role in autophagy or due to their engagement in the previously described depression-related pathways. In fact, it has been hypothesized that many of the effects of pharmacological autophagy inducers erroneously were ascribed to this degradative process, because several autophagy regulatory proteins exert functions beyond autophagy [124]. It is also plausible that there is overlap in the action of the membrane reorganizing machinery required for autophagy and synaptic function. In other words, the effect on membrane dynamics might be more important than the effects on protein homeostasis through autophagy.

### Inflammation

Several studies have found that MDD and a dysregulation of the inflammatory process are associated in a bidirectional pathway (“cytokine theory of MDD”) [125]. Immune cells mediate inflammation as an essential mechanism to maintain homeostasis by recognizing cell damage and aiding in tissue repair [126]. However, a sustained immune response such as in infection, malignancy, or autoimmune disease may result in depression [126]. Indeed, a heightened inflammatory response is linked to MDD. Specific proinflammatory cytokines and their receptors associated with MDD include interleukin (IL)-6, tumor necrosis factor (TNF)-α, IL-1β, IL-2, IL-2 receptor, IL-4, IL-10, the IL-1 receptor antagonist, the transforming growth factor-β, and C-reactive protein (CRP) [127, 128]. Proinflammatory cytokines also correlate with MDD symptom severity [129] and CRP with treatment-response [130].

There are many proposed mechanisms contributing to inflammation in MDD. The inflammasome pathway is an important source of proinflammatory cytokines [131], which can be activated in response to elevated levels of damage-associated molecular patterns (DAMPs) and other stress molecules, resulting in the activation of IL-1β and IL-18 [132]. DAMPs associated with depression include the high mobility group box-1 [133, 134], extracellular ATP [134], and circulating cell-free mitochondrial DNA [135]. Other inflammation-inducing factors include oxidative and nitrosative stress, psychosocial stress, poor diet, physical inactivity, obesity, smoking, and altered gut permeability [136]. Peripheral immune cells may also enter the central nervous system (CNS) through the blood-brain barrier (BBB), lymphatic vessels, or direct extravasation into the tissue [126]. Damage and loss of astrocytes in the frontal and limbic areas of the brain are also associated with MDD, contributing to BBB dysfunction and neuroinflammation [137, 138]. With increased BBB permeability, activated microglia can recruit monocytes to the brain via chemokines and produce interleukins that can further activate inflammation. Microglial function is controlled by the toll-like receptor pathway via recognition of DAMPs by microglia, and therefore chemokine production. Accordingly, increased levels of chemokine (C-X-C motif) ligand (CXCL) 4, CXCL7, and CXCL8 have been found in depression [139].

Clinical trials provide further evidence of the role of immune dysregulation in MDD. A meta-analysis of randomized controlled trials with patients who received anti-inflammatory therapy reported less depressive symptoms, higher remission, and a lower severity for all therapies [140]. Another mega-analysis found that patients who received immunological drugs targeting one of 7 mechanisms (IL-6, TNF-α, IL-12/23, CD20, COX2, BLγS, p38/MAPK14) had a significant improvement in depression, with the antidepressant effect being higher in the immunotherapy aimed at IL-6, IL-12, and IL-23 [141].

#### Link to synaptic activity

Mechanisms linking inflammatory pathways to synaptic activity include proinflammatory cytokines modulating the expression of the NMDA and AMPA receptor subunits and decreasing AMPA receptor phosphorylation, ultimately affecting glutamatergic synapses and processes related to LTP [142–144]. Indeed, cytokine-mediated synaptic plasticity is associated with cognitive function in MDD patients [144, 145]. Inflammatory cytokines also activate the enzyme indoleamine 2,3-dioxygenase (IDO) and thereby decrease the synthesis of 5-HT [146] (cf. “The monoamine theory” and “Metabolome/kynurenine pathway”). Further, activated microglia have been shown to irreversibly oxidize cofactors needed for the biosynthesis of monoamines [147] (cf. “The monoamine theory”). Finally, inflammation also influences the expression of excitatory amino acid transporters in astroglial cells, ultimately affecting glutamate uptake from the synaptic cleft [148].

#### Relation to other pathways

The immune system is tightly interrelated with the neuroendocrine system, with glucocorticoids having both pro- and anti-inflammatory effects depending upon the context [149, 150]. For instance, glucocorticoids can increase the expression of the inflammasome NLR Family Pyrin Domain Containing 3 (NLRP3) and promote the cleavage and secretion of proinflammatory cytokines [151]. In turn, many circulating cytokines can activate the HPA axis and ultimately increase adrenocorticotropic hormone and glucocorticoid levels [149, 152]. Increased glucocorticoids may further promote endothelial damage and contribute to BBB disruption [143], thus amplifying microglial activation and inflammation. In addition, proinflammatory cytokines not only reduce the expression of neurotrophins, but also inhibit BDNF/TrkB signaling by interfering with TrkB phosphorylation [153]. Finally, inflammatory mediators that are increased in MDD can significantly interfere with mitochondrial oxidative phosphorylation and ATP production, ultimately leading to increased oxidative stress [154]. The resulting dysfunctional mitochondria, in turn, can also further amplify the inflammatory response if not adequately removed by the mitophagy process (suggested for MDD [155], cf. “Mitochondrial dysfunction and oxidative stress”).

### Mitochondrial dysfunction and oxidative stress

As the cellular “powerhouse”, mitochondria play fundamental roles by providing energy for all cell functions and by acting as an important mediator of multiple signaling pathways, including those linked to monoamines, inflammation, and neural plasticity [156]. The “mitochondria theory of depression” is supported by a wealth of findings linking depressive symptoms and MDD to rare mitochondrial disorders [156], altered mitochondrial structure and functions including decreased ATP production [156, 157], and disrupted mitochondrial dynamics (fusion, fission, mitophagy) [155].

Mitochondrial disruption also generates free radicals and oxidative stress. In MDD, oxidative and nitrosative stress markers are increased, while antioxidant capacity is decreased [158–160]. Moreover, positive correlations with illness duration suggest a progressive course of mitochondrial dysfunction and oxidative damage with the disease [161]. Therefore, the “oxidative stress hypothesis of depressive disorders” proposes oxidative stress as the cause of the altered brain structure in MDD [162]. Notably, reactive oxygen species (ROS) at normal levels are important signaling messengers with key roles in neuronal cell function; however, when in high levels and with low antioxidant concentrations, these molecules can be detrimental for neurons and LTP. Indeed, the brain is particularly vulnerable to the effects of free radicals and ROS. Increased oxidative stress can potentially lead to further mitochondrial damage, increasing apoptosis and ultimately contributing to inflammatory signaling [162]. Finally, a key role for mitochondria and oxidative stress in MDD is further supported by preclinical and clinical studies suggesting antidepressant effects of drugs targeting these systems [163–165].

#### Link to synaptic activity

**Fig. 4:**
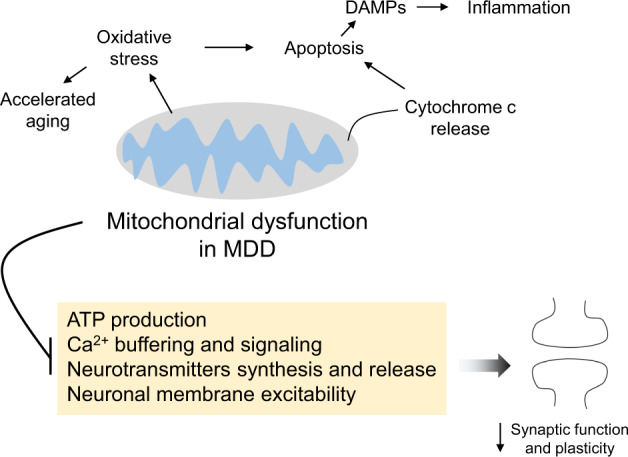
Theoretical model explaining the role of mitochondrial dysfunction and its effects on synaptic function in major depressive disorder (MDD). Disrupted mitochondria can lead to the activation of apoptosis and subsequent release of damage-associated molecular patterns (DAMPs), ultimately reinforcing inflammatory mechanisms. The resulting oxidative stress can also be associated with the accelerated aging phenotype consistently reported in MDD patients.

It is well-established that mitochondria support neurotransmission in several ways, including ATP production [166], Ca^2+^ buffering and signaling, synthesis of neurotransmitters [167] establishing and maintaining membrane excitability, and in the organization of synaptic vesicle pools and neurotransmitter release [168]. Mitochondria also produce oxygen and nitrogen species needed for synaptic plasticity, and activate caspases in dendrites to induce postsynaptic spine elimination involved in long-term depression [169] (Fig. 4). A very recent study reported not only changes in mitochondrial function in neural progenitor cells reprogrammed from fibroblasts of MDD patients compared to non-depressed controls, but also pronounced alterations of electrophysiological properties in neurons derived from induced pluripotent stem cells of MDD patients [170].

#### Relation to other pathways

The key cellular role of mitochondria comes with interdependency with numerous depression-relevant pathways. A biphasic effect of glucocorticoids is observed, where short-term exposure increases mitochondria’s B-cell-lymphoma 2 levels, calcium holding capacity, membrane potential, and oxidation, while long-term treatment at high levels can lead to mitochondrial toxicity [171]. Furthermore, dysfunctional mitochondria increase the production of proinflammatory cytokines [172], possibly mediated by the release of many DAMPs through mitochondrial outer membrane permeabilization [173]. These include, for instance, the mitochondrial DNA, which activates toll-like receptor 9 and the NLRP3 inflammasome, in addition to causing a type I interferon response [173]. Finally, neurotrophic signaling affects mitochondria, as exemplified by BDNF impacting mitochondrial mobility, distribution, and respiratory coupling which is at least partly required for its effect on neurotransmission [57, 174]. Like BDNF, proper mitochondria function impacts neuronal cell generation and death as low levels of ROS are neuroprotective and activate neuronal cell proliferation [67].

### Metabolome/kynurenine pathway

The combination of nutrients with the host’s metabolism [175] and gut microbes produces a rich variegation of chemicals (i.e., the metabolome) potentially impacting physiological processes at various levels. For the effects on brain function, some metabolites may cross the BBB and directly trigger relevant pathways, or may elicit a response in the periphery with repercussions on the brain, such as changing the hormone and cytokine profile in the blood or through neural effects linking to the brain [176, 177].

**Fig. 5:**
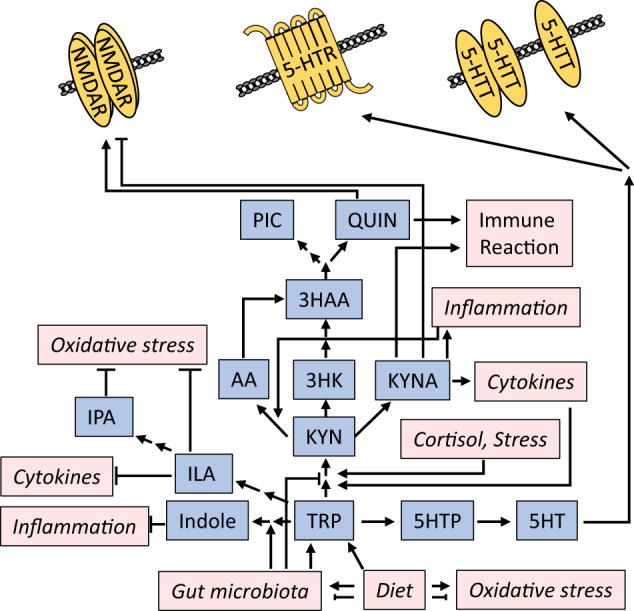
Kynurenine pathway exemplifying the role of metabolism and gut microbes in MDD. The conversion to serotonin takes place on the enterochromaffin cells of the intestinal mucosa, but also in the CNS. The kynurenine pathway produces numerous compounds that impact synaptic function either directly or indirectly through their influence on multiple systems, including immune function and oxidative stress. Conversely, these systems also shape the activity of several enzymes involved in the kynurenine pathway, in combination with diet and gut microbiota composition. TRP tryptophan, KYN kynurenine, 3HK 3-hydroxykynurenine (neurotoxic), KYNA kynurenic acid (neuroprotective), 3HAA 3-hydroxyanthranilic acid (neurotoxic), AA anthranilic acid, QUIN quinolinic acid (neurotoxic), PIC picolinic acid (neuroprotective), 5-HT serotonin, 5-HTR serotonin receptor, 5-HTT serotonin transporter, ILA indole-3-lactic acid, IPA indole-3-propionic acid.

Support for the relevance of this “gut-brain axis” includes gut microbiome changes in MDD [178]. Conversely, nutrient supplementation with probiotics or the Mediterranean diet elicits antidepressant effects in patients [179–181]. Causality between microbiome alterations and depressive-like behavior can also be inferred from experiments transferring fecal microbiota or specific bacteria [182–186]. The nutritional/microbiotic effects on the brain are described through links to established molecular pathways controlling synaptic function [187]. Thus, we focus on an eminent example, the pathway of kynurenine, which is a metabolite of the essential amino acid tryptophan (Fig. 5). Together with carbohydrate metabolism, tryptophan is one of the earliest nutritional links to depression first reported more than 60–80 years ago [188].

#### Link to synaptic activity

The conversion of tryptophan into the neurotransmitter 5-HT provides an obvious link to synaptic function and depression [189]. However, tryptophan is metabolized predominantly through the kynurenine pathway [190], which produces both neurotoxic (e.g., quinolinic acid) and neuroprotective (e.g., kynurenic acid) metabolites [190–192]. Kynurenic acid directly acts at the synapse as a glutamate receptor antagonist by binding to the glycine binding site [193]. In contrast, quinolinic acid is a glutamate receptor agonist [194]; it enhances release of glutamate and inhibits glutamate re-uptake by astrocytes [195, 196].

#### Relation to other pathways

Kynurenic acid is a potent antagonist of peripheral and central nicotinic acetylcholine α-7 receptors, which are linked to cytokine production, inflammation, and the immune reaction [197, 198], and are considered a potential drug target for depression treatment [197–199]. The kynurenine pathway is intertwined with numerous depression pathways, for example inflammation and immune cell activity, acute, chronic mild, and early-life stress [200–202], oxidative stress and mitochondrial function [203, 204], and BDNF signaling [205, 206] (Fig. 5).

## Integrative model of MDD neurobiology

The molecular pathways and theories selected for this review have been repeatedly conceptualized as unique and separate entities. Today, it is broadly accepted that these multiple pathways are not orthogonal, i.e., they are significantly interconnected. Nevertheless, there is some debate as to where the first causal disturbance may originate before involving other pathways, which we briefly discuss for mitochondria and stress.

### Mitochondria as initial disturbance?

Associations between mitochondrial genetic variations, cognitive function, and depression [170, 207] has prompted some authors to suggest mitochondrial dysfunction as the initiator of a chain of molecular events precipitating MDD. In fact, mitochondrial damage can ultimately cause the activation of apoptotic pathways, as previously evidenced in peripheral and brain samples of MDD patients [208, 209]. Apoptotic events may eventually contribute to the activation of the immune system and lead to the chronic low-grade inflammatory status seen in MDD [210]. However, in addition to mitochondrial damage, many other stimuli and mechanisms also excite the inflammatory phenotype of MDD, including a direct effect of oxidative and nitrosative stress, the microbiome-gut-brain axis, and many environmental factors highly prevalent in patients [136]. Other downstream mechanisms may originate from dysfunctional mitochondria or other stimuli, as well. For instance, oxidative stress can impact many pathways such as BDNF signaling, neuroplasticity, and cognition [211]. It can further cause DNA damage [212, 213], alter DNA methylation [214, 215], and induce accelerated aging [216], as reported for MDD [217].

### Stress as initial impact?

Chronic stress and HPA axis dysfunction, which are frequently proposed as primary players in the development of MDD, are linked to downstream effects that might be elicited through alternative pathways. These include mitochondrial alterations and dysfunction [218, 219], as well as apoptosis [171], immune activation, and inflammation. Glucocorticoid resistance, as seen in many MDD patients, has also been previously associated with increased inflammatory markers, supporting the hypothesis of a tight cross-talk between stress and inflammation in the disorder [220]. Alternative pathways may also further downstream effects of stress, including the contribution of inflammation to BBB disruption, which facilitates leakage of immune molecules into the CNS. This can ultimately induce microglial activation, impair hippocampal neurogenesis, and directly impact brain structure and function. Originating from stress or not, immune molecules can also stimulate IDO and thereby activate the kynurenine pathway, contributing to a reduction in hippocampal structure and volume in MDD [221].

Altered myelination is increasingly recognized as an important factor in both the etiology and treatment of MDD, and is another example of the difficulties in unequivocally proving the initial triggers [222]. Through enhancing conductivity along neuronal axons [223], myelin and myelin-producing oligodendrocytes are obvious candidates for mechanisms of brain diseases in general. Several studies found pronounced alterations in myelination and oligodendrocyte lineage cells in depression and animal models thereof [222]. Even though not typically conceptualized in pathways, myelin and oligodendrocytes are known to be affected by stress and by several other factors such as neurotransmitters, neurotrophins, cytokines, ROS, epigenetic factors, intestine microbiome, among others [222, 224]. Further, oligodendrocytes shape neuronal function in many ways beyond myelination; the importance of oligodendrocytes and myelination in MDD is corroborated by their response to antidepressant treatment [222].

### Multitude of interrelated pathways

The emerging role of polyamines in MDD etiology and treatment is another example of how interrelated the different pathways are [225]. Polyamines such as spermidine, spermine, putrescine and agmatine are short, aliphatic amines that impact several pathways and synaptic activity by a variety of mechanisms; the impacted molecules and pathways include almost all systems mentioned in this review, e.g., Na^+^-, K^+^- and Ca^2+^-channels, 5-HT, NMDA, AMPA, kainate, nicotinic acetylcholine and H^+^-receptors, cAMP/PKA, MEK/ERK, PI3K/Akt, GSK3/CREB, PI3K-Akt-mTOR, oxidative stress, and BDNF/TrkB [225]. Polyamines are also important players in the stress response [226] and autophagy, which involves a unique post-translational modification of the eukaryotic translation initiation factor 5A requiring spermidine as an essential substrate [227]. Thus, the autophagy inducer spermidine is proposed as a therapeutic strategy in aging and neurological disorders [228, 229], and dietary polyamines are considered to promote health in general [230]. Accordingly, the antidepressant-like effects of polyamines have been explained by various mechanisms ranging from direct effects at the synapse to regulating pathways linked to synaptic activity, as alluded to above [231].

**Fig. 6:**
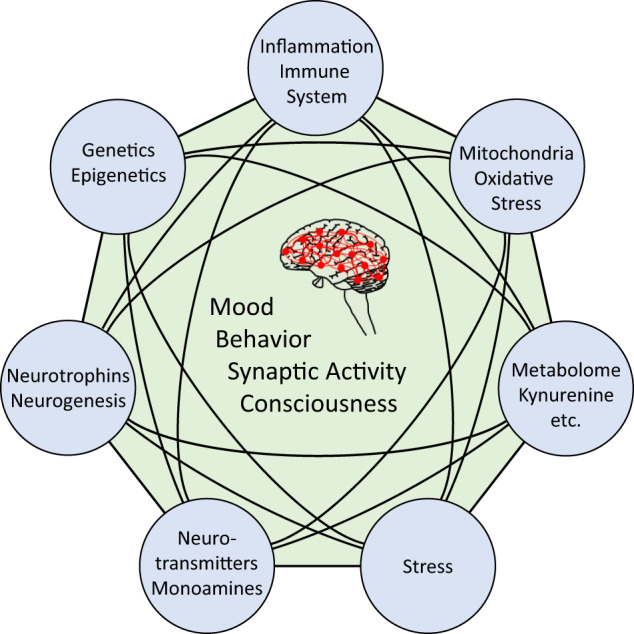
Multitude of molecular pathways and physiological systems determining mental health. The pattern of synaptic activity representing mood, behavior, consciousness, and thus also major depressive disorder (MDD), is governed by the concerted action of interrelated molecular pathways and physiological activities. This review exemplified molecular links between major systems contributing to the development of MDD. The relative contribution of each pathway varies between individual patients as a reflection of the high complexity of the disease.

Together, although the exact patterns of synaptic activities distinguishing health and disease in MDD are unknown, and arguably may never be resolved at a single synapse resolution, it is widely accepted that MDD may begin through several pathways and involves more mechanisms as the disease unfolds (cf. Fig. 6). Mirroring the clinical heterogeneity of MDD, not all patients present with the same neurobiological basis. For instance, significantly high inflammation is not found across all patients [232], and different levels of baseline inflammatory status have been shown to influence the patients’ treatment responsiveness [233]. This variation also applies to the other pathways selected here. Behavior and synaptic activity very likely rest on more than these pathways (Fig. 6), and the high interconnectedness between them challenges the concept to approach this complexity through pathway descriptions. For both clinical research and practice, much hope rests on using information on biological heterogeneity to better characterize clinical heterogeneity in MDD, and thus stratify patients for treatment and investigation. Regardless of the original stimuli that activate the chain of multi-pathway reactions characteristic of MDD, we submit that they may all converge to disarrayed synaptic activity by affecting the production and release of neurotransmitters, membrane excitability, dendritic spine elimination, among other mechanisms.

## Conclusions and outlook

A complete understanding of the associations of the multiple molecular pathways with MDD may be an unrealistic expectation. Nevertheless, evidence should be noted supporting their effects on key neuronal and synaptic functional measures not only for MDD (Fig. 6), but also for other major psychiatric disorders, such as bipolar disorder and schizophrenia. This suggests that studying endophenotypes across diagnostic boundaries rather than in MDD per se is more informative. Yet, the complex associations of the pathways pose a challenge to identify single actionable targets for new drug developments.

It might be advantageous to target more than one pathway with a single compound. The high biological heterogeneity of MDD across patients calls for the application of novel drugs, possibly in combination with established treatments to approach the goal of personalized medicine. When tapping into the wealth of correlative data from observational studies for choosing novel targets, a critical step will be the distinction between alterations causing the disease and alterations mounted by the organism to cope with the disease [234]. This challenge is further aggravated by the organization of pathways in feedback loops.

Remarkable methodological progress pathed the way to obtain functional neuronal cell cultures from patients and controls via differentiation of induced pluripotent stem cells or trans-differentiation of fibroblasts [235–239]. The synaptic and circuit activities of these neurons correlate with disease and treatment response [170, 240, 241], suggesting them as useful cellular models to investigate mechanisms shaping synaptic activity and function, and to test novel antidepressant drugs acting on them, complementing animal models [242]. Further, despite overwhelming consensus on the necessity, we are just beginning to understand sex specificity in the molecular and pathway correlations in MDD [243]. Finally, since pathways into a disease may not simply be the reverse pathways out of a disease, much is expected to be learnt from deciphering resilience factors [244] and the still not entirely resolved molecular actions of antidepressants [245, 246].

A prime example for the latter is the discovery of the acidic sphingomyelinase (ASM) as a target of several antidepressants, which evolved from the observation that antidepressants, due to their chemical nature, accumulate in lysosomes [67, 247]. ASM cleaves sphingomyelin into ceramide and phosphocholine, thereby not only impacting the biophysical properties of the cell membrane, but also myelination, cell differentiation/proliferation and cell death and birth, in particular also in the CNS [247]. Importantly, ASM/ceramides are linked to synaptic activity and pathways that had been associated with MDD before: these include TRPC6 and thus growth cone guidance, spine morphology, dendritic outgrowth and neuronal survival [248], as well as PKC and regulation of tubulin, GSK-3β, and β-catenin [67]. Ceramides are altered in MDD [249, 250] and several antidepressants at therapeutic concentrations are functional ASM inhibitors [67, 247]. These studies substantiate the relevance of this approach, and of the ASM/ceramide system in particular, justifying intensified efforts with the prospect for improved MDD treatment.
